# A cultural look at moral purity: wiping the face clean

**DOI:** 10.3389/fpsyg.2015.00577

**Published:** 2015-05-12

**Authors:** Spike W. S. Lee, Honghong Tang, Jing Wan, Xiaoqin Mai, Chao Liu

**Affiliations:** ^1^Department of Marketing, Rotman School of Management, University of TorontoToronto, ON, Canada; ^2^State Key Laboratory of Cognitive Neuroscience and Learning, IDG/McGovern Institute for Brain Research, and Center for Collaboration and Innovation in Brain and Learning Sciences, Beijing Normal UniversityBeijing, China; ^3^Department of Psychology, Renmin University of ChinaBeijing, China

**Keywords:** morality, purity, embodiment, metaphor, face, culture

## Abstract

Morality is associated with bodily purity in the custom of many societies. Does that imply moral purity is a universal psychological phenomenon? Empirically, it has never been examined, as all prior experimental data came from Western samples. Theoretically, we suggest the answer is not so straightforward—it depends on the kind of universality under consideration. Combining perspectives from cultural psychology and embodiment, we predict a culture-specific form of moral purification. Specifically, given East Asians' emphasis on the face as a representation of public self-image, we hypothesize that facial purification should have particularly potent moral effects in a face culture. Data show that face-cleaning (but not hands-cleaning) reduces guilt and regret most effectively against a salient East Asian cultural background. It frees East Asians from guilt-driven prosocial behavior. In the wake of their immorality, they find a face-cleaning product especially appealing and spontaneously choose to wipe their face clean. These patterns highlight both culturally variable and universal aspects of moral purification. They further suggest an organizing principle that informs the vigorous debate between embodied and amodal perspectives.

## Introduction

Across human societies, bodily purity is intertwined with morality. For example, ritual purification of the physical body symbolizes moral purification in all major religions around the world, from baptism of Christianity and Mikvah of Judaism, to ablution of Islam and Buddhism, to bathing in the Ganges of Hinduism and Amrit of Sikhism. In cross-national surveys, purity concerns constitute one of the foundations of moral intuition on all continents (Graham et al., 2011). Physiological and neural studies further show that physical contamination and moral violation alike activate the same facial muscles (for oral–nasal rejection; Chapman et al., 2009; Cannon et al., 2010) and overlapping brain networks (Moll et al., 2005; Borg et al., 2008), indicating biological underpinnings of the link between impurity and immorality. These observations lead one to expect that this psychological phenomenon, “moral purity” (Douglas, 1966; Zhong and Liljenquist, 2006; Williams et al., 2009), is universal. Is it?

We suspect it is and it isn't, depending on the theoretical level of universality under consideration (Norenzayan and Heine, 2005). The mere *existence* of the notion of moral purity is likely to be universal, given the pancultural and panreligious moralization of bodily purity. Beyond this minimal level of existential universality, however, the scientific literature is in the dark about whether different cultures are comparable or incomparable in the moral *functions* of purification (e.g., guilt reduction; Zhong and Liljenquist, 2006) and the effective *forms* of purification (e.g., washing hands, rinsing mouth; Lee and Schwarz, 2010). Although there is a rapidly growing body of experimental evidence for the diverse consequences of physical cleansing on moral emotion, judgment, and behavior (e.g., Schnall et al., 2008; Liljenquist et al., 2010; Zhong et al., 2010; Helzer and Pizarro, 2011; Huang et al., 2011; Ritter and Preston, 2011; Cramwinckel et al., 2013a,b; Xu et al., 2014), this work is completely silent on issues of universality, because all the data have come exclusively from Western samples. On the other side of the world, cleansing may have different (or the same) moral functions, and even the same moral consequences may result from different (or the same) effective forms of cleansing.

Drawing on theoretical perspectives on morality, culture, and meaning representation, we predict a distinct form of moral purification in the East. To maintain moral order, societies often invoke culturally significant meanings such as face, honor, and dignity (Leung and Cohen, 2011). Mental representations of these meanings can be grounded in bodily states and sensorimotor modalities (Barsalou et al., 2003), such as the head-high, chin-up, straightback posture of honor (IJzerman and Cohen, 2011). Among East Asians, in particular, the face is a culturally highlighted body part that represents the public image of the self. It is highly prominent in their sociomoral discourse, to the point where theorists refer to them as *face cultures*. Distinct from honor and dignity cultures, members of face cultures are strongly avoidant of “losing face,” which happens when they are seen acting selfishly, disloyally, or inappropriately; and they are keen on “gaining face” by moving up the social hierarchy so that others “give face” to them (Ho, 1976; Leung and Cohen, 2011). Given the face's prominent role in moral regulation and its chronic salience, face-cleaning may have particularly potent moral effects in an East Asian context, more so than in a Western context, implying a culture-specific form, but culture-general function, of moral purification.

To experimentally investigate this possibility, we first conducted a pilot study using the culture-priming approach (Oyserman and Lee, 2008). We sampled Chinese-Canadian biculturals, primed either Chinese or Canadian culture, and tested if face-cleaning effects on guilt depended on which culture was salient.

## Does face-cleaning alleviate guilt most effectively when an East Asian culture is salient?

In a 2 (Chinese- vs. Canadian culture-prime) × 2 (face- vs. hands-cleaning) between-subjects design (see Table 1 for sample characteristics), participants first saw the photos of six objects and places that were either characteristically Chinese (e.g., ping pong racket, soy sauce, Shanghai) or Canadian (e.g., ice hockey stick, maple syrup, Toronto), and were asked to use five words to describe the culture (Hong et al., 2000). Then all participants recalled and reported in detail a personal experience of doing something highly unethical. Next, under the pretense of a product evaluation survey, they were given an antiseptic wipe to evaluate and were either asked to try it on their face or on their hands.

**Table 1:** **Sample characteristics of all experiments**.

**Experiment**	***N***	**Age *M* (*SD*)**	**Background**	**Language of materials**
Pilot	105 (53 female)	21.64 (3.40)	Students born in China, Taiwan, or Hong Kong, and studying in a large university in Canada	English
1	95 (52 female)	22.20 (1.92)	Students in a large university in northeast China	Chinese
2	73 (47 female)	21.29 (2.05)	Students in a large university in northeast China	Chinese

Finally, all participants were asked, “How do you feel about the unethical behavior you described earlier?” They rated their feelings on seven items [0 = *Not at all (emotion)*, 10 = *Very (emotion)*]. *Guilty* and *regretful* were the items of interest; they are self-conscious emotions (Lewis, 2000) of relevance to face as one's public self-image (Ho, 1976). They were embedded among filler items (*sad, calm, angry, excited, anxious*), none of which is a self-conscious emotion. Guilty and regretful ratings were highly correlated [*r*_(105)_ = 0.77, *p* < 0.001] and averaged as the dependent measure of immoral feelings.

As predicted, participants who cleaned their face felt less immoral if they had been primed with Chinese (*M* = 3.98, *SD* = 2.45) than with Canadian culture (*M* = 5.38, *SD* = 3.00), planned contrast *t*_(101)_ = 1.80, one-tailed *p* = 0.04. This pattern did not emerge among participants who cleaned their hands; instead, they showed a non-significant trend of feeling less immoral if they had been primed with Canadian (*M* = 4.12, *SD* = 2.96) than with Chinese culture (*M* = 5.18, *SD* = 2.47), *t*_(101)_ = 1.46, *p* = 0.15. In short, the remorse-reducing effect of face-cleaning, but not of hands-cleaning, was contingent upon a salient East Asian culture [cleaning modality × primed culture *F*_(1, 101)_ = 5.33, *p* = 0.02].

These pilot results are consistent with our prediction that wiping the face clean exerts stronger moral influence in an East Asian context. To test it more rigorously, we conducted an experiment with several goals in mind. First, to provide a neutral baseline for comparing the face- and hands-cleaning effects, we added a no-cleaning condition. Second, we included measures of immoral feelings prior to the cleaning manipulation to ensure that any cleaning effect would not be an artifact of pre-existing emotional differences between cleaning conditions. Third, going beyond immoral feelings, we examined guilt-driven prosocial behavior to see if actual behavior would be affected by physical cleansing in a culture-specific way. If cultures are identical in their effective forms of purification, then the absolving effects of hands-cleaning among Westerners—as consistently demonstrated in prior research—should generalize to East Asians. But if cultures have different effective forms of purification, then hands-cleaning may not affect East Asians' moral behavior, whereas face-cleaning may.

## Does face-cleaning in particular reduce guilt-driven prosociality among East Asians?

In a single-factor design with three (face- vs. hands- vs. no-cleaning) between-subjects conditions, participants first recalled and reported an unethical experience as in the pilot study (except that the instructions were presented on screen and the experience reported into a microphone rather than on paper). Then they rated their current feelings on the 15-item State Shame and Guilt Scale (e.g., “I want to sink into the floor and disappear”; 1 = *not feeling this way at all*, 5 = *strongly feeling this way*; Marschall et al., 1994). Next, they were given a skincare wipe allegedly for product evaluation and were asked to either try it on their face or hands or simply examine it. Afterwards, participants completed a filler arithmetic task that ostensibly concluded the experiment.

Upon “completion,” participants were asked if they would be willing to volunteer for another graduate student who was in desperate need of data but running out of budget. Whether they volunteered to help was the dependent measure. In this paradigm, helping would increase with guilt (Zhong and Liljenquist, 2006), and refusing to help would indicate less guilt.

Post-experimental probing revealed that one participant was familiar with moral purity research and suspicious about the experiment's purpose; two were interrupted by phone calls and quitted early; two failed to recall any unethical experience. They were excluded from analysis (final *N* = 90).

Face-cleaning participants were four times more likely than no-cleaning participants to refuse to help [33.3 vs. 6.7%; *Wald − test*_(1)_ = 5.52, *p* = 0.02, 95% CI (0.06, 0.45)]. In contrast, hands-cleaning participants were not significantly different from no-cleaning participants in their refusal to help [16.7 vs. 6.7%; *Wald − test*_(1)_ = 1.37, *p* = 0.24, 95% CI (−0.07, 0.28)]. This pattern could not be explained by pre-existing differences in immoral feelings because the three groups reported comparable levels of shame and guilt prior to the cleaning manipulation [*M*_face−cleaning_ = 3.02, *SD*_face−cleaning_ = 0.39; *M*_hands−cleaning_ = 2.95, *SD*_hands−cleaning_ = 0.39; *M*_no−cleaning_ = 3.00, *SD*_no−cleaning_ = 0.38; *F*_(2, 87)_ = 0.26, *p* = 0.77]. In short, among our East Asian participants, face-cleaning reduced guilt-driven prosocial behavior; contrary to all prior findings among Westerners, hands-cleaning did not.

The data so far suggest face-cleaning to be a culturally distinct form of purification, with consequences on guilt and prosociality. Is it such an effective coping mechanism that East Asians would even spontaneously wipe their face clean in the wake of their immorality? We tested this in a final experiment by observing people's cleaning behavior. In addition, we measured their desire and willingness to pay for a facial cleanser among a variety of consumer products, to verify if their urge to cleanse was specific to the face.

## Does immorality specifically elicit face-cleaning desire and behavior among East Asians?

In a single-factor design with two (unethical vs. ethical recall) between-subjects conditions, participants first recalled and reported an experience of being either highly unethical or highly ethical. Three participants failed to report any unethical experience and were excluded from analysis (final *N* = 70). Then in an ostensibly unrelated consumer survey, they saw 20 products (Table 2), including facial cleanser, cleaning products for other specific body parts (e.g., hand wash, mouthwash), for the body in general (e.g., towel, soap), and for objects (detergent, washing powder), as well as non-cleaning products (e.g., battery, stapler). Participants indicated the desirability of each product (1 = *completely undesirable*, 7 = *extremely desirable*) and their willingness to pay for it (in Chinese ¥), which served as the first set of dependent measures.

**Table 2:** **Desirability of and willingness to pay (WTP) for each product as a function of unethical vs. ethical recall in Experiment 2**.

**Product**	**Desirability**				**WTP, log-transformed**			
	**Unethical *M* (*SD*)**	**Ethical *M* (*SD*)**	***t***	***p***	**Unethical *M* (*SD*)**	**Ethical *M* (*SD*)**	***t***	***p***
**Facial cleanser**	**4.63 (1.54)**	**3.86 (1.54)**	**2.10**	**0.04**	**1.26 (0.22)**	**1.15 (0.18)**	**2.22**	**0.03**
Hand wash	3.63 (1.61)	4.17 (1.67)	−1.38	0.17	0.85 (0.34)	0.96 (0.18)	−1.56	0.12
Hand moisturizer	4.17 (1.76)	3.77 (1.66)	0.98	0.33	0.82 (0.25)	0.81 (0.29)	0.04	0.97
Mouthwash	3.20 (1.98)	2.69 (1.80)	1.14	0.26	0.81 (0.43)	0.83 (0.36)	−0.18	0.86
Toothpaste	5.34 (1.41)	4.77 (1.54)	1.62	0.11	0.86 (0.21)	0.81 (0.19)	1.16	0.25
Towel	5.20 (1.30)	4.66 (1.45)	1.65	0.10	0.96 (0.24)	0.92 (0.21)	0.69	0.49
Antiseptic wipe	4.06 (1.63)	4.00 (1.73)	0.14	0.89	0.48 (0.23)	0.48 (0.21)	−0.09	0.93
Soap	3.57 (1.77)	4.00 (1.78)	−1.01	0.32	0.79 (0.44)	0.84 (0.38)	−0.48	0.63
Detergent	4.80 (1.69)	4.54 (1.36)	0.70	0.49	1.12 (0.25)	1.09 (0.17)	0.53	0.60
Washing powder	3.89 (1.86)	4.34 (1.45)	−1.15	0.26	0.69 (0.19)	0.77 (0.21)	−1.57	0.12
Mint	4.20 (1.35)	4.17 (1.42)	0.09	0.93	0.87 (0.19)	0.87 (0.16)	−0.04	0.97
Post-it Note	3.57 (1.60)	3.63 (1.33)	−0.16	0.87	0.55 (0.19)	0.55 (0.16)	−0.04	0.97
Stapler	3.74 (1.34)	3.63 (1.44)	0.35	0.73	0.88 (0.26)	0.84 (0.23)	0.74	0.46
Pen	4.83 (1.67)	4.74 (1.48)	0.23	0.82	0.49 (0.12)	0.49 (0.16)	0.06	0.96
Chewing gum	4.09 (1.50)	4.00 (2.00)	0.20	0.84	0.51 (0.18)	0.47 (0.17)	1.08	0.28
Snickers	3.06 (1.92)	3.83 (1.82)	−1.72	0.09	0.52 (0.27)	0.60 (0.21)	−1.26	0.21
CD	3.20 (1.61)	3.31 (1.69)	−0.29	0.77	0.71 (0.37)	0.80 (0.35)	−0.99	0.33
Candy	3.89 (1.71)	4.26 (1.69)	−0.92	0.36	0.46 (0.22)	0.46 (0.15)	0.12	0.91
Battery	3.71 (1.92)	3.43 (1.70)	0.66	0.51	0.58 (0.25)	0.50 (0.16)	1.52	0.13
Wafer	4.29 (2.03)	4.49 (1.82)	−0.43	0.67	0.88 (0.30)	0.86 (0.28)	0.21	0.84

*Bold text indicates the product of primary theoretical interest*.

Next, as a basic check of whether participants associated the cleaning products with various body parts in ways we expected, they rated the extent to which each bodily cleaning product was related to the face, hands, mouth, feet, and hair (1 = *completely unrelated*, 7 = *highly related*). Not surprisingly, participants across conditions rated the facial cleanser as highly related to the face, hand wash and hand moisturizer to the hands, mouthwash and toothpaste to the mouth, and other cleaning products to multiple body parts (see online Supplemental Material).

Finally, participants were given a skincare wipe for product evaluation and asked to try it (without being told how). Unbeknownst to them, which body part they started wiping was unobtrusively recorded by the experimenter. It turned out all participants started wiping either their face or hands, which served as a dichotomous behavioral measure.

As expected, recalling an unethical rather than ethical experience increased participants' desire for the facial cleanser [*M*_unethical_ = 4.63, *SD*_unethical_ = 1.54; *M*_ethical_ = 3.86, *SD*_ethical_ = 1.54; *F*_(1, 68)_ = 4.41, *p* = 0.04, *d* = 0.51, 95% CI (0.04, 1.51)] and willingness to pay for it [log-transformed *M*_unethical_ = 1.26, *SD*_unethical_ = 0.22; *M*_ethical_ = 1.15, *SD*_ethical_ = 0.18; *F*_(1, 68)_ = 4.93, *p* = 0.03, *d* = 0.54, 95% CI (0.01, 0.21)]. No significant effect was found on any other product (Table 2), suggesting that for East Asians immorality may specifically potentiate the appeal of face-cleaning. Indeed, participants who recalled an unethical rather than ethical experience were twice as likely to spontaneously wipe their face clean [46 vs. 23%; χ^2^(1, *N* = 70) = 4.06, *p* = 0.04, φ = 0.24, 95% CI (0.01, 0.42)].

## General discussion

Moral purification is manifest in a culture-specific form, distinct from all prior findings in the West. Given East Asians' emphasis on the face as a representation of their public self-image, making their cultural background salient renders face-cleaning especially effective for alleviating guilt and regret about their past misdeeds. Wiping the face clean frees them from guilt-driven compensatory prosociality. Its effectiveness as a coping mechanism is further demonstrated by their specific interest in a face-cleaning product and face-cleaning behavior in the wake of their immorality.

### Implications for the cultural variability and universality of embodied metaphors

Comparing and contrasting these findings with all prior experimental data on moral purity suggests that it is a psychological phenomenon with both culturally variable and potentially universal aspects. At one level, the moral benefits of hands-cleaning are clearer among Westerners than East Asians, for whom face-cleaning works better (presumably because the facial modality is chronically salient; see next section). This pattern indicates potential cultural variability in the most effective forms of purification. At another level, immorality does elicit cleansing desire and actual cleansing does reduce guilt among East Asians as well. Even though the strongest claim of universality is to be withheld until worldwide experimentation (Norenzayan and Heine, 2005), finding that physical cleansing produces the same kind of affective, judgmental, and behavioral consequences among Easterners and Westerners alike—despite their substantially different values, beliefs, self-views, relational styles, cognitive, and perceptual foci (Oyserman et al., 2002)—reinforces the possibility that moral purification, while culture-specific in form, is culture-general in functions and existence.

Embodied metaphors such as Morality Is Purity, Affection Is Warmth, and Importance Is Weight may appear to be universal phenomena, given their supposed evolutionary origins (Williams et al., 2009) or pancultural presence in languages, taboos, or rituals (Douglas, 1966; Kövecses, 2000; Zhong and Liljenquist, 2006). Even if their existence is universal, however, their psychological functions or effective forms may still vary. The level at which these and other embodied metaphors fundamental to social life are culture-specific or culture-general remains a wide-open empirical question.

### Implications for embodied vs. amodal perspectives

Facial cleaning has particularly potent effects among East Asians, for whom the face is a chronically highlighted modality. Juxtaposing these and prior findings suggests a possible organizing principle: moral purification is specific to the salient modality. The salience can be chronic, as in a face culture. Or it can be temporary, as when saying vs. doing something unethical makes people want to clean their “dirty mouth” vs. “dirty hands” (oral vs. manual modality; Lee and Schwarz, 2010).

This principle of modality salience is compatible with models that ground knowledge in modality-specific systems (Barsalou et al., 2003). It may be generalized to predict that embodied metaphors exert the strongest influence via whichever modality is most salient in a situation, to an individual, or for a culture. For example, moral effects of hands-cleaning are particularly powerful for individuals with obsessive-compulsive disorder who are chronically compelled to wash their hands (Reuven et al., 2013). Because the causal role of modality salience is predicted a priori by embodied models but only accommodated *post hoc* in amodal models, providing evidence for or against its robustness holds promise for informing the vigorous debate between embodied and amodal perspectives.

### Conflict of interest statement

The authors declare that the research was conducted in the absence of any commercial or financial relationships that could be construed as a potential conflict of interest.
