# Supplementary material for: A cultural look at moral purity: wiping the face clean
Source: Front Psychol. 2015 May 12;6:577. doi: 10.3389/fpsyg.2015.00577 (PMC4428058; doi:10.3389/fpsyg.2015.00577)
Supplement: Supplementary file 1 [file Table1.DOCX]

1. **Supplementary Material**

**Table S1.** Perceived relations of bodily cleaning products to various body parts, showing comparable ratings across conditions (unethical vs. ethical recall) in Experiment 2.

|  |  | Relation to face | | | |  | Relation to hand | | | |  | Relation to mouth | | | |  | Relation to hair | | | |  | Relation to foot | | | |
| --- | --- | --- | --- | --- | --- | --- | --- | --- | --- | --- | --- | --- | --- | --- | --- | --- | --- | --- | --- | --- | --- | --- | --- | --- | --- |
| Product |  | Unethical *M* (*SD*) | Ethical *M* (*SD*) | *F* | *p* |  | Unethical *M* (*SD*) | Ethical *M* (*SD*) | *F* | *p* |  | Unethical *M* (*SD*) | Ethical *M* (*SD*) | *F* | *p* |  | Unethical *M* (*SD*) | Ethical *M* (*SD*) | *F* | *p* |  | Unethical *M* (*SD*) | Ethical *M* (*SD*) | *F* | *p* |
| Facial cleanser |  | 6.94 (0.24) | 6.94  (0.24) | 0.00 | 1.00 |  | 4.63 (1.82) | 5.09 (1.63) | 1.23 | .27 |  | 3.23 (2.29) | 3.24 (2.06) | 0.00 | .99 |  | 1.43 (0.92) | 1.37 (0.88) | 0.07 | .79 |  | 1.40 (0.81) | 1.34 (0.84) | 0.08 | .77 |
| Hand wash |  | 2.00 (1.28) | 1.86  (1.44) | 0.19 | .66 |  | 6.66 (1.11) | 6.91 (0.37) | 1.69 | .20 |  | 1.63 (1.22) | 1.69 (1.30) | 0.04 | .85 |  | 1.43 (0.78) | 1.31 (0.72) | 0.41 | .53 |  | 2.06 (1.66) | 1.89 (1.45) | 0.21 | .65 |
| Hand moisturizer |  | 3.03 (1.76) | 2.57  (1.70) | 1.22 | .27 |  | 6.83 (0.62) | 6.94 (0.24) | 1.05 | .31 |  | 1.80 (1.39) | 1.74 (1.15) | 0.04 | .85 |  | 1.26 (1.04) | 1.26 (0.66) | 0.00 | 1.00 |  | 2.57 (1.93) | 2.43 (1.96) | 0.09 | .76 |
| Mouthwash |  | 1.89 (1.41) | 1.60  (0.98) | 0.97 | .33 |  | 1.40 (0.98) | 1.43 (0.95) | 0.02 | .90 |  | 6.86 (0.49) | 6.26 (1.60) | 4.51 | .04 |  | 1.17 (0.51) | 1.14 (0.43) | 0.06 | .80 |  | 1.14 (0.55) | 1.11 (0.40) | 0.06 | .81 |
| Toothpaste |  | 1.97 (1.38) | 2.00  (1.28) | 0.01 | .93 |  | 1.89 (1.26) | 1.94 (1.16) | 0.04 | .84 |  | 6.74 (0.56) | 6.23 (1.63) | 3.12 | .08 |  | 1.20 (0.53) | 1.26 (0.66) | 0.16 | .69 |  | 1.20 (0.41) | 1.31 (0.72) | 0.67 | .42 |
| Towel |  | 6.86  (0.36) | 6.66  (1.08) | 1.08 | .30 |  | 6.63 (0.65) | 6.51 (0.89) | 0.38 | .54 |  | 5.29 (2.08) | 5.37 (2.07) | 0.03 | .86 |  | 6.26 (1.17) | 6.31 (1.13) | 0.04 | .84 |  | 5.17 (2.07) | 5.43 (1.87) | 0.30 | .59 |
| Antiseptic wipe |  | 6.51 (1.01) | 6.46  (0.85) | 0.07 | .80 |  | 6.51 (0.95) | 6.46 (0.66) | 0.09 | 0.77 |  | 5.00 (2.43) | 5.31 (1.78) | 0.38 | .54 |  | 1.80 (1.41) | 1.31 (0.63) | 3.46 | .07 |  | 2.63 (1.93) | 2.03 (1.51) | 2.11 | .15 |
| Soap |  | 5.71 (2.07) | 5.91 (1.84) | 0.18 | .67 |  | 4.71 (2.11) | 5.00 (1.86) | 0.36 | .55 |  | 3.14 (2.16) | 3.00 (2.06) | 0.08 | .78 |  | 1.66 (1.00) | 1.60 (1.01) | 0.06 | .81 |  | 2.31 (1.59) | 1.97 (1.45) | 0.89 | .35 |
